# Si Nanowires: From Model System to Practical Li-Ion Anode Material and Beyond

**DOI:** 10.1021/acsenergylett.4c00262

**Published:** 2024-03-17

**Authors:** Syed Abdul Ahad, Tadhg Kennedy, Hugh Geaney

**Affiliations:** † Department of Chemical Sciences, University of Limerick, Limerick V94 T9PX, Ireland; ‡ Bernal Institute, University of Limerick, Limerick V94 T9PX, Ireland

## Abstract

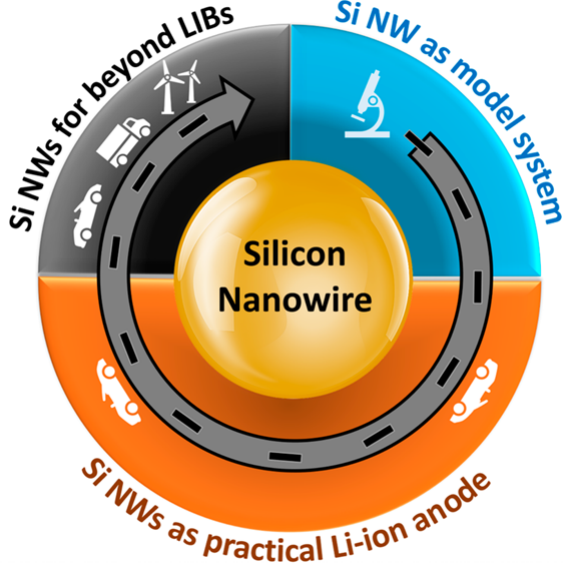

Nanowire (NW)-based anodes for Li-ion batteries (LIBs) have been under investigation for more than a decade, with their unique one-dimensional (1D) morphologies and ability to transform into interconnected active material networks offering potential for enhanced cycling stability with high capacity. This is particularly true for silicon (Si)-based anodes, where issues related to large volumetric expansion can be partially mitigated and the cycle life can be enhanced. In this Perspective, we highlight the trajectory of Si NWs from a model system to practical Li-ion battery anode material and future prospects for extension to beyond Li-ion batteries. The study examines key research areas related to Si NW-based anodes, including state-of-the-art (SoA) characterization approaches followed by practical anode design considerations, including NW composite anode formation and upscaling/full-cell considerations. An outlook on the practical prospects of NW-based anodes and some future directions for study are detailed.

LIBs are a revolutionary energy storage technology that underpin important applications from portable electronics to electric vehicles (EVs) and large-scale stationary energy storage.^1^ The development of LIBs has been inextricably linked to the pursuit of high-performance active materials, with the first commercial LIB iteration in 1991 followed by more than three decades of active materials research. This materials timeline is punctuated by significant optimization of the cathode active materials, with fewer deviations on the anode side, with graphite remaining the dominant material over many decades. Higher-capacity anode active materials are an integral component of the next generation of higher energy density (ED) LIBs, with research interest largely divided between metallic anodes (e.g., Li) and those based on Si. The ability of Li to form Li-rich alloys with Si has been known for many decades,^2^ according to the general equation *x*Li^+^ + *x*e^–^ + Si → Li_*x*_Si (0 ≤ *x* ≤ 3.75), with large values of “*x*” leading to high specific capacities.^3,4^ The value of *x* here is linked to the electrochemically achievable composition of the Li_*x*_Si alloy at room temperature, rather than the higher Li-containing phase Li_4.4_Si, which is a high–temperature phase.^4^ However, the volume expansion of Si during lithiation means that significant optimization of the active material is required to enable reversible alloying/dealloying over repeated cycles without pulverization or degradation of the active material. This has led to a range of different Si nanostructures in the literature, which have unlocked long-term cycling stability compared to micrometer-sized Si particles.^5−8^ Complementing this, a range of active material modification approaches, including structure regulation, interfacial engineering, binder design and optimization, and electrolyte composition control, have been widely studied to enhance the practical prospects of Si-based anodes.^9−11^

A particularly notable (and widely studied) morphology of Si for LIBs is 1D Si NWs as pioneered by Cui and colleagues in 2008.^12^ Their binder-free architecture involved Si NWs grown directly on a stainless-steel current collector using a gold (Au) seed layer and allowed practical specific capacities in excess of 3000 mAhg^–1^ to be delivered. The 1D morphology of the NWs and electrochemically assisted structural evolution into an interconnected matrix meant that large-scale material pulverisation that had previously prevented the use of Si in LIBs could be avoided, spawning a wave of interest in “NW batteries”. The wide variety of established synthesis approaches for Si NW growth (e.g., chemical vapor deposition (CVD) and solution based approaches) and well-understood growth mechanisms (vapor liquid solid, VLS; vapor solid solid, VSS; and solution liquid solid, SLS) meant that Si NW-based anodes could be widely formed and quickly became a hot topic in LIB anode materials development. Si NWs have also become a “go-to” model system for examining critical battery-related phenomena such as solid electrolyte interphase (SEI) formation^13,14^ and alloying/dealloying driven expansion/contraction^15^ and are also ideal materials for a variety of operando analytical techniques.^16^

Particular focus in the early stage of this development (up to the mid 2010s) was on the formation of NWs with increasing materials complexity,^17,18^ spanning compositional heterostructures,^19^ alloys, and related materials like Ge NWs.^20,21^ These approaches demonstrated the ability of various NW-based anodes to sustain long-term alloying/dealloying reactions with Li (as compared to micrometer-sized Si particles) to deliver far beyond SoA specific capacity values. It should be noted that this Perspective is not a comprehensive review on Si NW growth methods, which have been dealt with in a large number of excellent review articles in the last two decades.^22−26^ Si NWs offer distinct advantages compared to 0D nanoparticles: (i) Their micrometer scale lengths allow the formation of dense entangled networks, bringing structural rigidity and the ability to adhere to CCs far better than particles.^12^ (ii) Si NWs with micrometer scale lengths allow the growth of hierarchical structures, increasing the effective electrochemical surface area.^19^ (iii) NWs have been shown to transform into unique active material matrices upon cycling, which leads to cycling stability to hundreds of cycles.^27^ (iv) NWs directly grown from (or on) CCs are highly attractive from an ED perspective as they remove the requirement for conductive additives and binders (such as the NW architecture commercialized by Amprius). (v) The high aspect ratios of NWs increase electrode–electrolyte contact, promoting material utilization during electrochemical reactions. (vi) NWs in general shorten the ionic transport pathways, since diffusion length is proportional to the diffusion time, which is beneficial for achieving high rate capability.^28^

Despite the promise of Si NWs for practical systems, the directly grown approach raises challenges in achieving practically relevant areal capacities, which will be discussed in depth in the following sections. As the obvious alternative to directly grown materials, conventional slurry-based Si NW anode formation has typically been challenging from a synthesis/upscaling perspective. These challenges include production of gram to kilogram scale powders, which is difficult with CVD-based growth methods. This has been circumvented by solution-based approaches,^29^ while different sacrificial hosts (e.g., sodium chloride (NaCl)^30^) and electrochemically active growth hosts (e.g., graphite^31^) have also been used to increase the achievable mass of Si NW active materials. Given the trend in EV LIBs toward the incorporation of Si as a capacity-boosting additive in graphite-based anodes, there is now particular interest in the formation of graphite/Si composite active materials, which may circumvent some of the challenges related to material upscaling. In parallel investigations, the use of different NWs (metals, silicides, etc.) as electrochemically inactive hosts for active Si has also attracted attention, with the goal being to produce conductive architectures that can accommodate active Si and tolerate the active material expansion/contraction during cycling without anode pulverisation. These investigations have often led to demonstrations of areal capacity values that are compatible with commercial targets (typically >3 mAh cm^–2^);^32−34^ however, the additional mass added by the use of structured current collectors, alongside processability and manufacturing compatibility, must be considered when judging the practical potential of these systems.

In this Perspective, we examine key aspects of Si NW anode material development, as depicted in Figure 1. First, we discuss the use of Si NWs as a model system to examine fundamental lithiation/delithiation processes. Advanced characterization methods for the interrogation of crucial battery mechanisms are highlighted, with Si NWs being an ideal candidate for these investigations. We then discuss the development of directly grown anode materials based on Si NWs and coated amorphous(a)-Si on NW host structures, examining their relevant strengths and weaknesses and key areas for future development. These approaches focus on delivering high areal capacity, high initial Coulombic efficiency, active material swelling control, and long-term cycling stability by understanding and mitigating failure mechanisms.^9−11^ Building upon the requirement for upscalability and the trend toward Si/graphite composite material development in commercial EV cells, we examine the routes toward Si NW graphite composite material formation. We reflect on the key findings in recent years that have major implications for all Si-based anode materials in LIBs. Finally, we discuss recent investigations on beyond-LIB approaches that exploit the unique properties of Si NWs for Li metal, solid-state battery (SSB), and sodium (Na)-ion battery development. In total, the Perspective highlights the major progress made in Si NW development in the recent decade, with prospects for future directions and eventual commercialization.

**Figure 1:**
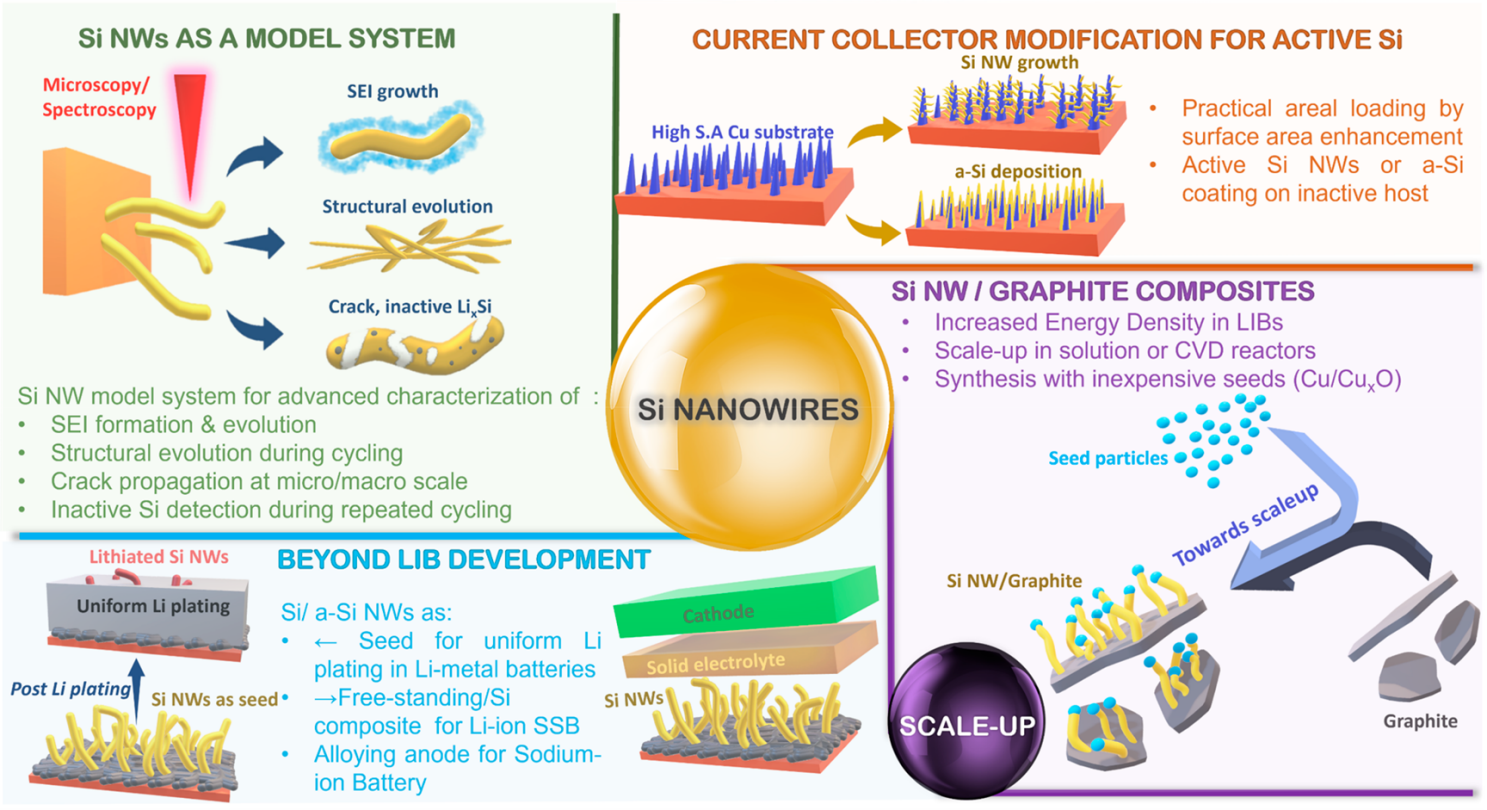
Schematic illustration demonstrating the scope of Si NWs in LIBs and beyond, as discussed in this Perspective.

## Si NWs as a Model System

**Figure 2:**
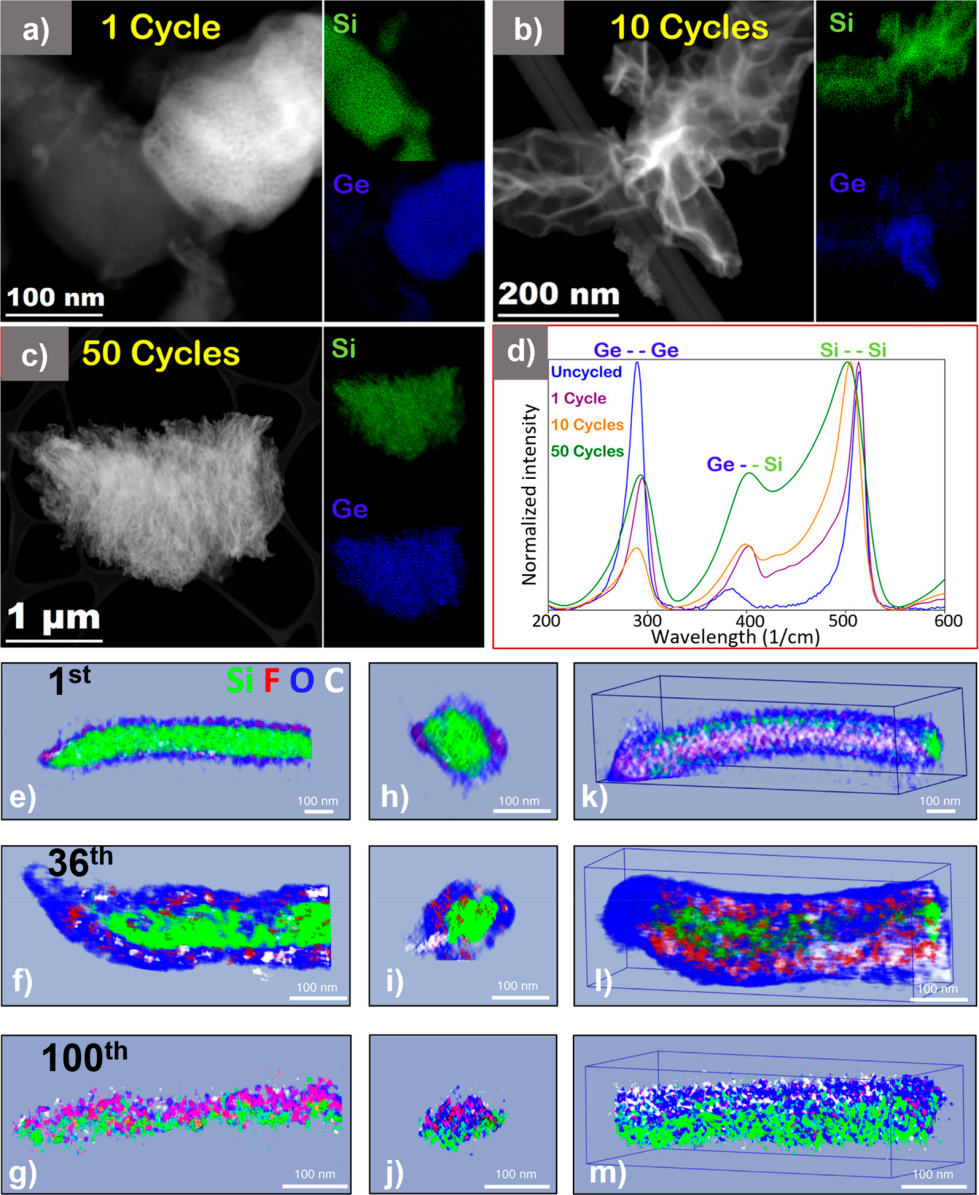
Structural evolution and EDX mapping of the evolution of Si–Ge heterostructures after the (a) 1st, (b) 10th, and (c) 50th cycle. (d) Corresponding Raman spectra of the pristine and cycled NW structures after 1st, 10th, and 50th cycle. ((a–d) Reproduced from ref (36). Copyright 2018 American Chemical Society.) (e–g) Segmented view parallel to the *x*-axis. (h–j) Segmented cross-sectional view. (k–m) 3D view of Si NW from 3D cryo STEM-EDS analysis after 1st, 36th, and 100th cycle. ((e–m) Reproduced with permission from ref (37). Copyright 2021 Springer Nature.)

Si NWs represent an ideal and longstanding platform for the investigation of the critical failure mechanisms of Si-based anodes using advanced characterization techniques such as electron microscopy and spectroscopy techniques, as they remove interference from binders and conductive additives.^35^ In our recent work,^36^ we used Si–Ge heterostructure NWs as a model system to track the compositional and morphological evolution of Li alloying anodes using dark-field scanning transmission electron microscopy (DF-STEM). Ex situ analysis was used to track the NW morphology from a compositionally segregated amorphous material (1st cycle, Figure 2a) into a porous structure with a maintained NW silhouette (10th cycle, Figure 2b) and finally into a compositionally homogeneous (SiGe alloy) mesh-like, active materials matrix (50th cycle, Figure 2c). This transformation was evident in Raman analysis, where an increased peak intensity linked to Ge–Si was noted (Figure 2d). Such structural transformation due to repeated volume changes during cycling is believed to be beneficial for the ionic and electronic transport within the active material. A similar NW evolution mechanism was revealed by cryogenic STEM in conjunction with elemental tomography to simultaneously track SEI evolution and Si changes during cycling^37^ (Figure 2e–m). The results suggest electrolyte permeation into voids and a progressive SEI formation. The structure therefore changes from a core–shell Si/SEI structure to a “plum-pudding” structure after extended cycling (Figure 2g,j,m). The analysis suggests thick SEI formation around the Si domains, thereby disrupting electronic contact with the active material, along with the formation of “dead” Si within the electrode. This leads to capacity decay due to active material loss, poor Coulombic efficiency (CE), and loss of Li inventory in full-cells as well. Although fluoroethylene carbonate (FEC) is known to create a hydrofluoric acid (HF)-resistant SEI layer^38^ which would be beneficial in resisting corrosion of Si, excessive buildup of any type of SEI-layer would be detrimental for cyclic performance as shown by cryo-TEM (combined with electrochemical testing) for the FEC-containing electrolyte.

Given the importance of Li inventory for Si-based LIB cells, additional methods are required to assess cyclable Li depletion during cycling. This loss of Li due to the formation of irreversible Li and SEI formation can be gauged using titration-gas chromatography^39^ (TGC). This technique involves electrochemical (de)lithiation of Si to develop an SEI layer and any irreversible Li_*x*_Si alloys, followed by immersion of the retrieved electrode in ethanol solvent.^40^ H_2_ gas evolution due to the reaction of ethanol with Li_*x*_Si alloys can be used to quantify this component in the anode. Since ethanol does not react with Li containing SEI components, the amount of Li trapped in the SEI layer components can be determined by subtracting from the total moles of Li measured from the electrochemical testing. 3D X-ray nanocomputed tomography (XRD-CT) is another powerful technique that should be more widely extended to Si NW-containing electrodes to determine the evolution and failure mechanism of these electrodes in the presence of binders and active material at the macroscopic scale. A study of Si nanoparticle-based electrodes showed that two degrees of lithiation have different effects in promoting Si agglomeration and nonuniform stress distribution during cycling.^41^ In this study, higher lithiation levels (2000 mAh g^–1^) induced early failure due to excessive volume expansion, delamination, and limited active material utilization compared to lower depth-of-discharge (DOD) levels (1000 mAh g^–1^). However, this effect has not been examined in the wide range of potential Si nanostructures, where different capacity fade mechanisms (or mitigations) may be linked to the active material morphology. Using advanced characterization techniques may finally allow the link between Si nanomorphology (NWs compared with nanoparticles or different architectures) and long-term cycling stability trends to be clearly explained. Moving beyond these fundamental considerations, the role of CCs in Si NW-based anodes is a critical aspect that requires further interrogation.

## Current Collector Modification for Active Si

Current collectors (CCs) within LIBs endure harsh operating conditions such as interactions with potentially corrosive electrolytes, active material swelling during cycling, etc., making them a critical and often overlooked aspect of stable battery operation. Copper (Cu) and aluminum (Al) have long been adopted as the CCs for anodes and cathodes, respectively, in LIBs.^42^ Though Al is cheaper and has much a lower density than Cu (2.6 g cm^–3^ vs 8.96 g cm^–3^), it alloys with Li (0.26 V vs Li/Li^+^) in the anode operational voltage window (0–1.5 V) and cannot be used as an anode CC.^43,44^ CVD enables active material growth directly on different types of CCs (e.g., stainless-steel (SS) foil, Nickel (Ni) etc.) and tunable loadings on the current collector. The pioneering work by Chan et al. used gold (Au) catalyst deposited on SS foil to grow Si NWs via high-temperature CVD;^12^ however, stainless-steel has a large weight penalty compared to Cu.^45^ Furthermore, the achievable mass loadings on planar CCs are often below what is required to achieve practical areal capacities, so significant focus has shifted to the development of high surface area current collectors to increase the Si NW loading and obtain practical areal capacities.

**Figure 3:**
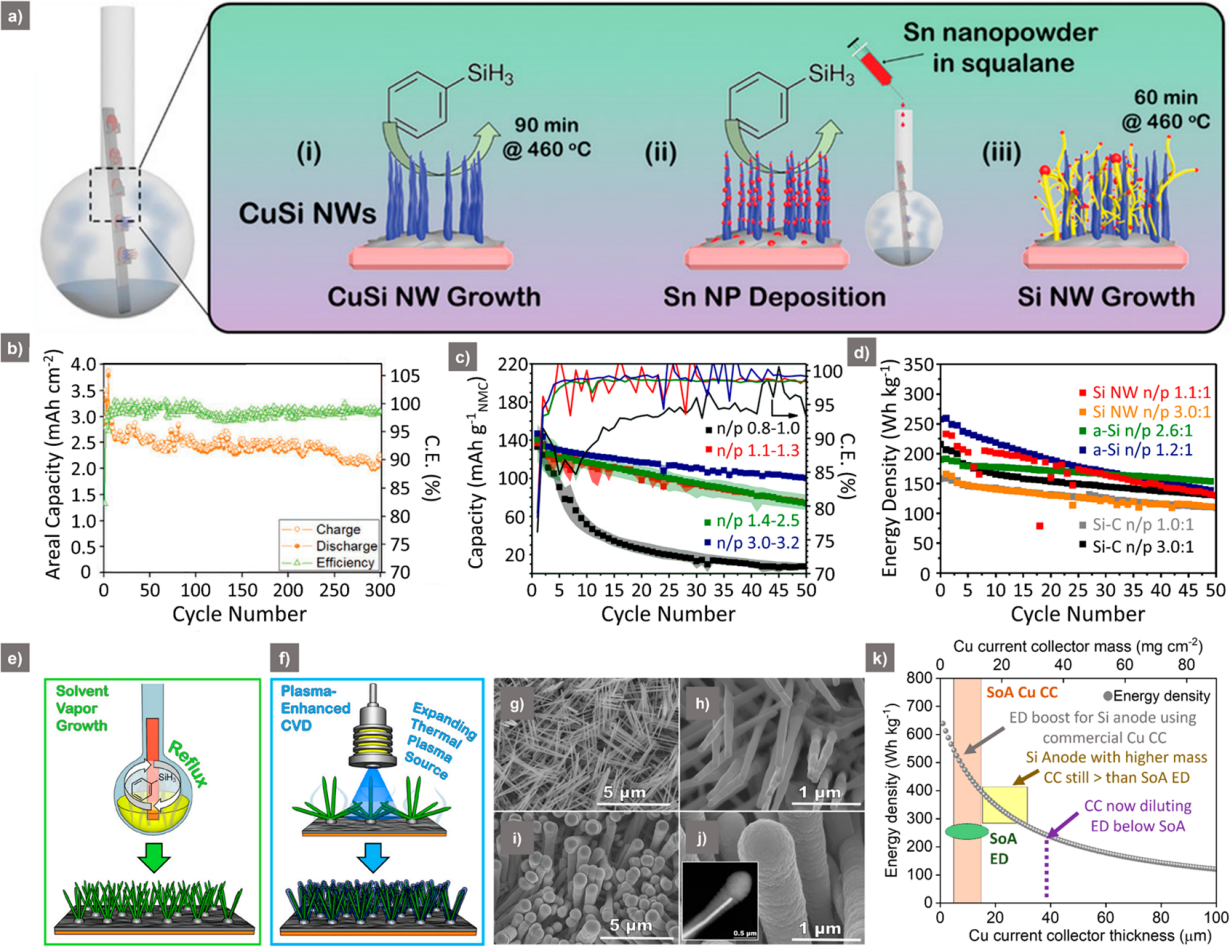
(a) Schematic of one-pot synthesis method of Si NWs grown on CuSi NWs. (b) Long-term cyclic performance and CE of Si/CuSi-Li with 1.60 mg cm^–2^ loading at 0.2*C*. ((a, b) Reproduced with permission from ref (34). Copyright [2021] John Wiley and Sons.) (c) Cyclic performance and corresponding CE of Si NW/NMC111 full-cell at various N/P ratios (0.8–3.2 V) at 0.2*C*. (d) Comparison of the cyclic performance of Si NW/NMC111, a-Si/NMC111, and Si–C/NMC111 at various N/P ratio cycles at 0.2*C*. ((c, d) Reproduced with permission under a Creative Commons CC BY 4.0 License http://creativecommons.org/licenses/by/4.0/, ref (55). Copyright [2020] IOP Publishing Ltd.) (e, f) Schematic of solvent-assisted CVD process to synthesize Cu_15_Si_4_ (CuSi) NWs and a-Si coating on CuSi NWs using magnetron sputtering. (g, h) Corresponding SEM images of Cu_15_Si_4_ (CuSi) NWs. (i, j) Corresponding SEM images of a-Si coated CuSi NWs with inset representing TEM image of CuSi core and a-Si shell. ((e–j) Reproduced from ref (58). Copyright 2019 American Chemical Society.) (k) ED vs Cu CC thickness graph representing change in ED with increase in thickness (weight) of Cu CC. For comparison, SoA energy density (ED) range (green) of graphite-based LIB full-cells and commercially used Cu CC thickness (orange) is also given (assuming a 4 mAh cm^–2^ a-Si coating and a 3.8 mAh cm^–2^ NMC cathode).

One such example is the transformation of a Cu CC into a high surface area, electrochemically inactive Cu_15_Si_4_ (CuSi) NW network.^46^ The glassware-based method for CuSi NW growth has previously enabled the growth of Si and Ge NWs using low-cost catalysts (Indium, In; Tin, Sn; Zinc, Zn)^47−51^ and is a versatile system for LIB anode material synthesis. These CuSi NW-decorated CCs were used as a host for the growth of Sn-seeded Si NWs^34^ (Figure 3a), with the high CuSi NW surface area allowing a significant increase in active mass loadings up to 1.6 mg cm^–2^. In comparison, planar SS/Cu foils were only capable of accommodating 0.1–0.4 mg cm^–2^ following similar reactions.^13,52,53^ This significant increase in the Si NW loading on the CuSi NW substrate led to a high initial areal capacity of 4 mAh cm^–2^, stabilizing at 2.2 mAh cm^–2^ after 300 cycles at 0.2*C* (Figure 3b). Postcycling analysis showed a dense network of Si NW-derived active material mesh, which was well adhered to the CuSi NW substrate. This structured nature of CC is significant, as it ensured that the electrical contact of active material was maintained to allow maximum capacity retention during long-term cycling. Other than NWs, structured CuSi foams have also been coated with Si NWs^54^ via a two-step process using the same liquid-based precursor approach. The foam was able to host significantly higher Si NW loadings (>1 mg cm^–2^) compared to planar CCs, delivering an areal capacity of 2.0 mAh cm^–2^ after 550 cycles.

Though numerous publications report the synthesis and cycling performance of Si NW anodes in half cells (vs Li metal), the literature on full-cell demonstrations studying the effect of N/P ratio (negative to positive electrode capacity ratio) is still scarce.^33,47,54,55^ Such studies are important to demonstrate the effect of SEI formation during initial cycles, cyclic stability, and prevention of Li plating during long-term cycling, especially when a limited amount of Li reservoir is present in the lithiated cathodes vs Si NW anodes. A relevant study demonstrated the effect of varying N/P ratios (0.8–3.2) in Si NW – lithium nickel manganese cobalt oxide (NMC811) full-cells on their cyclic performance (Figure 3c).^55^ In the case of N/P = 0.8, the capacity quickly dropped, suggesting a Li inventory loss in the form of Li plating on the Si anode. The performance improved with an increase in the N/P ratios, with N/P = 3.2 demonstrating the best performance, mainly due to the partial utilization of the Si NW anode. Even though the full-cell performance was decent, the Si anode excess required for a high N/P ratio (i.e., 3.2) results in poor ED of the full-cell, making such a configuration commercially not viable. In comparison to Si NWs, partially lithiated amorphous (a)-Si/NMC showed better initial ED. This trend was valid at low (1.1) and high (2.6–3.0) N/P ratios, demonstrating that a-Si can be an attractive alternative to crystalline Si in certain cell configurations (Figure 3d).

To this end, various studies have shown the promise of a-Si as a high-capacity coating on high surface area NW-decorated CCs.^56−58^ CuSi NW networks with NW diameter of ∼80–100 nm grown on a Cu CC via CVD (Figure 3e,g,h) were further coated with a-Si using plasma-enhanced CVD.^58^ The a-Si with a mass loading of 0.2 mg cm^–2^ was conformally coated around the CuSi NWs, with the NW morphology still visible afterward (Figure 3f,i,j). The electrochemical cycling demonstrated initial specific capacities of 3500 mAh g^–1^, which stabilized to 2000 mAh g^–1^ after 200 cycles at 0.2*C*. The high surface area substrate also facilitated a specific capacity of ∼1300 mAh g^–1^ at 5*C*. Postcycling analysis demonstrated that the a-Si converted to a porous structure, with embedded CuSi NWs, ensuring good electrical contact between the active material and CC. However, the effect of increased a-Si loading to commercially relevant levels on the CuSi CC (and other nanostructured CCs) remains a largely open question in the literature.

Though binder-free Si NW (or a-Si) coating on high surface area CCs can be a path to higher ED, the mass and/or thickness of CCs used in many reports are usually missing, with little effort made to calculate ED (this is particularly the case where only half-cell testing is carried out). The ED plot in Figure 3k focuses on the impact of the mass of planar Cu CC (assuming a 4 mAh cm^–2^ a-Si coating and a 3.8 mAh cm^–2^ NMC cathode^59^) on ED. For the industrially used Cu CC thickness range of 5–15 μm, a high ED range of 400–545 Wh kg^–1^ can potentially be achieved (values on the curve shaded in orange). However, once the Cu CC thickness is increased to 31–37 μm, the ED of the a-Si/NMC full-cell decreases to ∼250–275 Wh kg^–1^, which is equivalent to that of SoA graphite/NMC full-cell. Therefore, while designing CCs for active Si coatings (or any other high specific capacity material that is grown from the CC), the density and thickness of the CC must be taken into consideration, to ensure that the high specific capacity active material can be translated into practical ED enhancements. This points to a critical current collector mass target of <40 mg cm^–2^, which is particularly important in the case of porous CCs (which may have significant volumetric ED penalties compared to planar CCs if the degree of porosity is high). The use of thick foils (including Cu and stainless-steel) will not be useful for practical systems, as heavy current collectors can completely negate the benefit of the high specific capacity active coating. Furthermore, attempts should be made to coat active material on both sides of the designed CC to maximize the ED gains of binder-free anodes. Thus, researchers are encouraged to report on the mass and thickness of nanostructure current collector hosts in future studies and ideally push toward full-cell testing as a central focus of this research.

## Si NW/Graphite Composites

**Figure 4:**
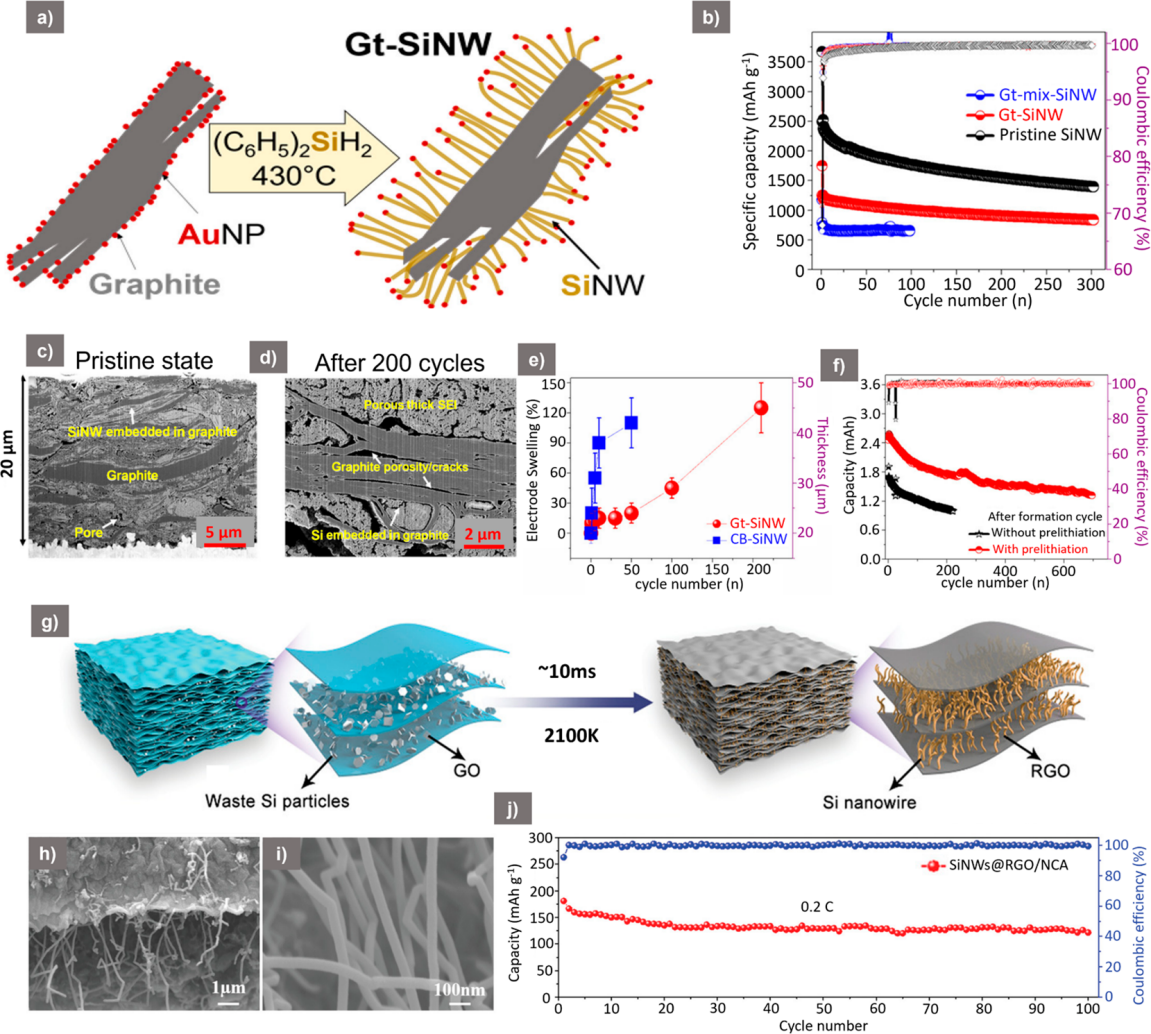
(a) Schematic of the synthesis procedure of Si NW growth on graphite flakes. (b) Cyclic performance and CE of Gt-mix-Si NW, Gt-Si NW composite, and pristine Si NWs at 0.2*C*. (c, d) Cross-sectional FIB-SEM of SiNW-Gt composite before cycling (pristine state) and after 200 cycles. (e) Comparison of electrode swelling (%) vs cycle number of Gt-Si NW and CB-Si NW composites cycled at 0.2*C*. (f) Cyclic performance of Gt-SiNW/NMC full-cell with and without prelithiation of Gt-SiNW anode at 0.2*C*. ((a–f) Reproduced from ref (31). Copyright [2020] American Chemical Society.) (g) Schematic of rGO/Si NWs anode by electrothermal shock process using GO and waste Si (WSi) as starting material. (h, i) SEM images of rGO-Si NWs anodes at different magnifications. (j) Cyclic performance and corresponding CE of SiNWs@rGO-NCA full-cell at 0.2*C*. ((g–j) Reproduced with permission from ref (68). Copyright [2021] John Wiley and Sons.)

While binder-free coating of Si NWs (or a-Si) is being investigated by a large number of researchers and some companies (e.g., Leyden Jar, Amprius), significant efforts have also been directed toward improving graphite-based full-cells by the incorporation of Si to generate Si/graphite composites.^60−62^ In this respect, Si NW/Graphite (Gt)^31^ composites were formed via a low-temperature CVD process using Au catalyst/Gt mixture and diphenylsilane as Si precursor, with potential for scale-up of the process (Figure 4a). This approach generated a Si NW content of 32% in the Si/Gt composite and a notably enhanced specific capacity compared to a simple graphite/Si NW mixture, while pristine Si NWs delivered the highest specific capacity among all of the compositions tested (Figure 4b). Examination of the pristine Si NW/Gt electrode showed Si NWs embedded between the graphite flakes and also pores within the slurry prepared electrode (Figure 4c). However, after 200 cycles, pores and cracks appeared in the graphite flakes with Si agglomerates still visible along with a thick, porous SEI layer (Figure 4d). Nevertheless, the whole structure was mechanically robust, with controlled swelling during repeated cycling when compared to a reference Si NW/carbon black (CB) structure (Figure 4e). While the SiNW/CB material swelled by 55% after just 5 cycles with subsequent exfoliation from the current collector, the SiNW/Gt composite swelled by up to 50% only after 100 cycles. This led to reduced electrode pulverization and indicated a regulation of the volume expansion during repeated cycling. The SiNW/Gt-NMC full-cell characterization demonstrated an ED of 414 Wh kg^–1^, with a capacity retention of 70% after 300 cycles (Figure 4f). This study highlights a number of key considerations for Si/Gt compositions. The significant volume expansion of Si compared to graphite will likely lead to a “sweet spot” in terms of the Si content, where the faster capacity decay associated with a higher Si anode is no longer offset by an increase in the starting ED. In the formation of composite electrodes, porosity to accommodate the volumetric expansion of Si will likely improve lab-scale capacity retention testing but must be considered in the context of practical anodes, where porosity leads to diminution of volumetric ED (Wh L^–1^) and an increased electrolyte requirement.

Commercial 18650 cylindrical cells tested with Si particles/Gt composites (5% Si) revealed that capacity loss from the Si/Gt anode was higher compared to the capacity loss from the cathode component during progressive cycling.^63^ SEM analysis suggested that even with 5% Si, macrocracks appeared in the anode which can cause excessive SEI formation and electrolyte depletion, indicated by increased fluorine (F) content around the agglomerated Si particles. As a general guideline, it is suggested that while designing Si NW and Si NW/Gt composites, the direct contact between active material and electrolyte should be minimized to mitigate interfacial side reactions. Various strategies to integrate functional coatings on Si NWs, including the use of electrolyte additives, ionic liquids, and self-healing polymer binders to develop a robust SEI layer and mitigate unnecessary volume expansion/material pulverization, have been examined and discussed elsewhere.^64^ However, special attention should be given toward the scalability of these strategies and their integration, particularly into SiNW/Gt composite anode containing cells which can be seen as the stepping stone technology to fully Si anode-based LIBs. Furthermore, the compatibility of any of these approaches with the corresponding cathode should be assessed from the outset to avoid incompatibility issues at the full-cell testing level.

A missing factor in most development studies for Si NW or Si NW/Gt electrodes is the effect of the electrolyte volume on the cyclic stability. A study on the effect of electrolyte volume on the performance of a 15 wt % Si/Gt-NMC pouch cell suggested that if the amount of electrolyte was not adjusted according to the ratio of electrolyte/pore volume of the electrodes and separator, it resulted in unreacted regions of active material, coupled with Li plating on the anode.^65^ It was thus suggested that in Si/Gt-NMC pouch cells, a minimum electrolyte/pore volume ratio of 3.1 was required to achieve decent electrochemical performance without significantly increasing the cell resistance and avoiding Li plating during cycling. However, this factor is not universal to various types of Si or Si NW/Gt composites, since the pore volume will vary with the properties of the composites produced (distribution of NW lengths and diameters, pore size, surface area, electrode processing, tap density etc.). In-depth characterization is required to determine the influence of these factors specifically for an NW morphology. Therefore, techniques such as nanocomputed tomography should be employed to study in detail the pore volume and surface area of the electrodes and their evolution with progressive cycling to determine the optimal volume of electrolyte required.

In a different vein for Si NW/C composites, the use of recycled materials for the generation of battery electrodes has gained much interest to reduce concerns over battery raw materials and the associated environmental impacts of mining.^66,67^ One such example includes the synthesis of SiNW/reduced graphene oxide (rGO) electrodes using waste-Si (WSi)^68^ powder via joule heating (Figure 4g). The overall process involves flash heating of WSi/GO composite films at high temperature (2100 K) in just 10 ms, followed by quenching to generate catalyst-free, micrometer-sized long Si NWs within the rGO sheets (Figure 4h,i). The Si content could be varied up to 76% by varying the WSi/GO ratio. The electrochemical performance measured an initial high Coulombic efficiency of 89.5% even at a high Si loading (3.67 mg cm^–2^), attributed to the conductive graphitized matrix of rGO as well as its high electronic and ionic nature promoting stable SEI formation. The SiNWs/lithium nickel cobalt aluminum oxide (NCA) full-cell performance showed a promising initial ED of 651.6 Wh kg^–1^ which stabilized to 454.7 Wh kg^–1^ (126.3 mAh g^–1^) after 100 cycles at 0.2*C* (Figure 4j). It would be extremely interesting to see if the SiNW/rGO composite materials could be processed into larger pouch cells and still deliver similar ED values as they would be well beyond those of SoA LIBs. Scaling-up of Si-based composites and subsequent pouch-cell level (or cylindrical/prismatic if achievable) testing is critical to ensure that the results can be brought to a higher technology readiness level (TRL) and ultimately commercialization. A number of key considerations include determining the feasibility of an upscaled synthesis process, identifying a practical slurry composition (binder/conductive additive ratios, solvents etc.) and electrode characteristics (mass loading, thickness and porosity), as well as optimizing the electrochemical cycling conditions (preconditioning, N/P ratio balance, and optimal asymmetric cycling conditions).

So far, few reports have been published on the design of scale-up reactors for Si NWs for LIB applications. One reported approach is based on the use of iodine gas reacting with low-grade micro Si particles to produce SiI_4_ gas, which then decomposes at 900 °C to form kinked Si NWs (diameter of 20 nm) which agglomerate into micro clusters.^69^ The decomposition of SiI_4_ results in the regeneration of I_2_ gas, which can be reused. After cost-benefit analysis, the Si produced from SiI_4_ was slightly cheaper than that produced by using SiH_4_ gas. The electrochemical cycling also showed promising activation of the Si NW clusters, exhibiting a capacity retention of 83.6% after 1000 cycles at 0.5*C* in a half-cell configuration. Recently, much interest has been gained by commercial entities such as OneDBattery Sciences, which has produced large-scale SiNW/Gt composites via a CVD process using low cost Cu/Cu_*x*_O catalysts.^70−72^ Their Si/Gt composites containing 21% Si demonstrated a lithiation specific capacity of 1048 mAh g^–1^ in the first cycle, with an impressive initial CE of 92.8%. This means that only 22 kg of Si/Gt composite would be required, instead of 58 kg of graphite in a 75 KWh EV battery pack. Detailed electrochemical testing reports and product roll-out are awaited to fully gauge the potential of this material; however, these recent demonstrations indicate the ability of Si NW/C composites to move well beyond 300 Wh kg^–1^. Furthermore, the graphite used in commercial batteries costs $6/kWh (vs $2/KWh variable cost of adding Si NW to graphite),^73^ which should be considered as a benchmark for further development of high ED anodes for LIBs. Here it is worth mentioning that other than the SiNW/graphite composite developed by OneD Battery Sciences, companies such as Sila Nanotechnologies have developed proprietary nano-Si (0D) confined in a scaffold matrix,^74^ claiming that the material is 5× lighter than graphite with 20% higher ED. However, the Si content in this active material is unknown, while the product’s extensive electrochemical testing in real world application to gauge its full performance is still awaited. Graphite Si NW/C may also serve as a stepping stone technology prior to widespread rollout of pure Si anodes, where the specific challenges associated with high-capacity alloying mode anodes (long-term processes like SEI, Li inventory loss, and cell swelling) can be gauged and addressed.

## Si NWs for Beyond-LIBs

**Figure 5:**
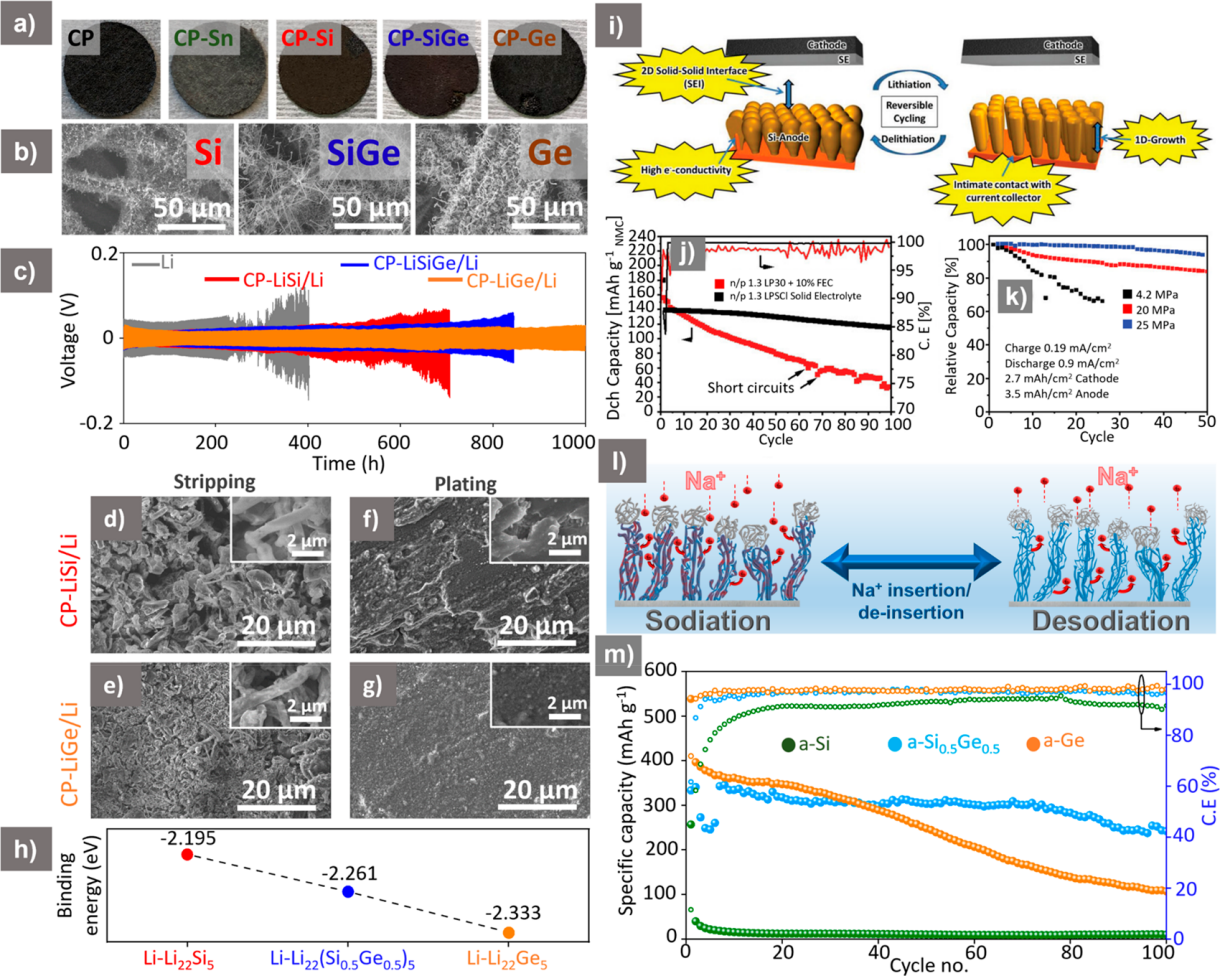
(a) Photographs of CP, Sn-CP, SiNW-CP, SiGe NW-CP, and Ge NW-CP electrodes after synthesis process. (b) SEM images of Si, SiGe, and Ge NWs grown on CP substrate. (c) Symmetric cell performance of Li, CP-LiSi/Li, CP-LiSiGe/Li, and CP-LiGe/Li anodes at 1 mA cm^–2^ and 1 mAh cm^–2^ current density and areal capacity. SEM analysis of cycled CP-LiSi/Li and CP-LiGe/Li electrodes after (d, e) stripping and (f, g) plating after 50 cycles. (h) DFT calculations determining binding energy of Li with Li–Si, Li–SiGe, and Li–Ge phases. ((a–h) Reproduced with permission from ref (75). Copyright [2023] John Wiley and Sons.) (i) Schematic of using columnar a-Si deposited on dendritic Cu for ASSBs. (j) Cyclic performance and CE comparison of NMC/SE/col-Si full-cell and NMC/LP30 + 10%FEC/col-Si cycled between 2.0 and 4.0 V at 1 mA cm^–2^ where LP30 is 1 M LiPF_6_ in EC: DMC (50:50, v/v %). (k) Capacity retention profile of NMC/Li_6_PS_5_Cl/col-Si pouch-bag based full-cells at various stack pressures of 4.2, 20, and 25 MPa. ((i–k) Reproduced with permission from ref (78). Copyright [2020] John Wiley and Sons.) (l) Schematic of the sodiation/desodiation process of mesh-type alloying NWs. (m) Cyclic perfromance of a-Si, a-SiGe, and a-Ge NWs in a NIB cycled between 0.005 and 2.0 V at 50 mA g^–1^. ((l, m) Reproduced with permission from ref (79). Copyright [2021] Royal Society of Chemistry.)

The use of Si NWs is not restricted to alloying anodes for LIBs. Significant interest has been shown in the use of Si NWs in other battery systems such as Li-metal,^75,76^ Li-solid-state,^77,78^ and Na-ion batteries.^79,80^ Li-metal anodes face problems such as dendrite formation and “dead” Li formation, which can be controlled by various strategies,^81^ including Li-alloy scaffold formation, which act to reduce the Li nucleation overpotential.^82−84^ The Li alloy can act as a nucleation point for subsequent Li deposition,^85,86^ and depending on the Li-ion diffusivity of the Li alloy, can promote uniform Li deposition. In this context, the NW morphology can play a significant role in Li metal anodes as it will increase the electrode surface area, decreasing the Li-ion flux density and therefore increasing the homogeneity of Li-ion flux distribution, promoting uniform Li deposition and stripping.^87,88^ Based on these concepts, we have examined Si, Si_0.5_Ge_0.5_ (SiGe), and pure Ge NWs grown on a 3D carbon paper (CP) as lithiophilic hosts for Li-metal batteries.^75^ The 3D NW-CP substrates showed a change in color depending on the composition of the NW grown on the substrate (Figure 5a), with SEM analysis showing dense NW coverage, which acted as the lithiophilic sites for Li incorporation (Figure 5b). Fast Li incorporation (∼4s) was observed when NW-CP substrates were infilled with molten Li, demonstrating good lithiophilicity of the Si, SiGe, and Ge NWs. In addition to elemental Li, Li-rich alloys (Li_22_Si_5_, Li_22_(Si_0.5_Ge_0.5_)_5_, and Li_22_Ge_5_) were also formed, which acted as seed layers for uniform Li stripping/plating. The symmetric cell testing demonstrated improved cycling of all the NW-CP compositions compared to the pure Li symmetric cell (Figure 5c). Post cycling SEM analysis revealed somewhat nonuniform Li (though critically without significant dendrite formation) on the Si NW derived (CP-LiSi/Li) substrate in the plated state compared to the stripped state (Figure 5d,f). Comparatively, the Ge NW-derived (CP-LiGe/Li) substrate showed uniform Li stripping (revealing the underlying lithiated NWs) and plating (showing uniform Li coverage) after repeated cycling (Figure 5e,g). Density functional theory (DFT) calculations suggested an increase in binding energy with the increase in Ge content, with the highest binding energy obtained with the pure Li_22_Ge_5_/Li phase, consistent with the experimental performance obtained (Figure 5h). With this unique architecture, further work is required to improve the performance of Si NW-based lithiophilic hosts for Li-metal batteries considering Ge is an expensive alternative. However, this study clearly demonstrates the potential of Si NWs for Li metal anode applications, wherein the cyclable Li is in the form of Li metal. This would drastically reduce the required areal loading of Si NWs compared to an alloying mode-based operation, opening a path to realizing higher ED anodes. Future investigations need to focus on the total anode mass of these “hosted” metal anodes to ensure that the added mass from the current collector (consistent with the discussion in Figure 3k) does not dilute the ED of practical cells.

Si NW use has mostly been examined in Li-ion/Li-metal batteries; however, it has also gained some interest in solid-state batteries (SSBs) as well as alternative alkali-ion batteries (i.e., Na-ion). Recently, various types of Si, such as monolithic,^89^ microparticles,^90,91^ nanoparticles,^92^ amorphous films,^93,94^ and columnar a-Si,^78^ have been used with different solid electrolytes (SE), with demonstrations of exceptional performance even with micrometer-sized Si particles. However, the use of nanostructured Si and especially 1D Si morphologies^77,78^ in SSB is still in its infancy, and a complete mechanistic understanding of the interfacial reactions (especially SEI formation) is required. The use of columnar 1D a-Si directly deposited on a Cu CC in a Li-ion SSB has been reported (Figure 5i), which enabled intimate contact between the active Si and the dendritic Cu CC during repeated cycling.^78^ The full-cell performance of NMC-Li_6_PS_5_Cl (LPSCl)–Si with an N/P ratio of 1.3 and a Si areal capacity of 3.5 mAh cm^–2^, demonstrated 118 mAh g^–1^, equivalent to a capacity retention of 82%, at 1 mA cm^–2^ after 100 cycles (Figure 5j). Comparatively, a full-cell containing liquid electrolyte had an adverse performance, indicating poor SEI formation and progressive electrolyte depletion during cycling. Furthermore, the importance of the cell pressure in Si-based SSB pouch cells was also investigated. At low pressure (4.2 MPa), the interfacial contact between the Si anode and the SE was lost, resulting in poor cell performance (Figure 5k); however, the interfacial contact was maintained at higher pressure, culminating in a 95% capacity retention after 50 cycles at 25 MPa. At this stage, it is unclear if Si NWs have any distinct advantages over micrometer-sized Si within SSBs. It would be interesting to examine how the Si NW and Si NW/Gt composite perform within SSBs. Depending on the anode architecture, the Si NWs might be able to accommodate volume expansion better compared to other morphologies while minimizing the pressure differential at the Si-SE interface. However, these future investigations require detailed chemomechanical analysis of the Si NW-based SSB to identify these characteristics.

Na-ion batteries are gaining much interest due to the larger natural reserves of Na present in the earth’s crust compared to Li.^95^ Hard carbon is considered the most stable anode for sodium-ion batteries (NIBs) but has low specific capacity.^96^ Though Si has a high theoretical capacity of 725 mAh g^–1^ based on the formation of the NaSi phase, crystalline Si (c-Si) does not perform well due to the difficulty in Na alloy formation.^97^ Several reports suggest that a-Si has enhanced Na-ion diffusivity compared to c-Si (7.2 × 10^–10^ cm^2^ s^–1^ for a-Si^98^ vs 10^–22^ cm^2^ s^–1^ for c-Si^99^), and a number of studies have investigated the use of thin a-Si coatings and low material loadings to enable cycling.^80,98,100^ As established within LIBs, NWs offer advantages, such as reduced diffusion length and controlled volume expansion without pulverization, which can be a design option for NIB anodes. In our previous work,^79^ we illustrated Na-ion activation of amorphized, NW derived Si, SiGe, and Ge mesh-type structures. The alloying of these compositions increased the Na-ion diffusivity and decreased the diffusion lengths (Figure 5l). However, due to the poor Na ion diffusivity within a-Si, it did not cycle without the addition of Ge as an alloying element. Among the compositions tested, the Si_0.5_Ge_0.5_ alloy showed promising results, demonstrating 250 mAh g^–1^ after 100 cycles without the use of any conductive agent or binder (Figure 5m). Though pure Si NWs could not be cycled, the synergistic effect of Si and Ge highlights the importance of studying alloy chemistry to activate Si to its full specific capacity within NIB. Further design advances, including incorporating conductive coatings to improve Na-ion diffusivity, might be helpful in enhancing the performance of Si and SiGe NWs in NIBs.

## Perspectives on the Future of Si NWs for Energy Storage Applications

Si NWs have been widely investigated within LIB applications, with distinct focus on electrochemically active c-Si and a-Si coated on conductive NW hosts. Directly grown anodes can achieve practical areal loadings, but significant consideration must be given to minimize the addition of excess CC mass, which can dilute ED. Si/graphite composites are an emerging stepping stone anode composition that will likely enable EDs > 300 Wh/kg and serve as a testing ground for the full Si anodes of the future. Long-term aging analysis of commercial Si/C cells should serve as a critical feedback avenue for the development of pure Si anodes.

The depth of reports on Si NWs-based full-cells is currently not comprehensive enough to gauge the upscaled benefits of Si NWs compared to other Si morphologies (i.e., micrometer-scale or nanoparticle Si). It is therefore important to test the full-cell performance of the proposed Si NW structures/composites by pairing them with high areal capacity cathode systems while fairly reporting the N/P ratios used in the full-cells. Prelithiation strategies (if employed) should be clearly reported to reflect the entire Li inventory in the cell. It is only within that context that useful ED values that allow comparison with existing SoA cell chemistries and Si alternatives can be generated.

To date, the majority of the studies conducted for Si NWs are based on coin cell-type configurations. However, to gauge the true potential of pure Si NW anodes and Si NW/Gt composites, large-scale pouch-cell type configurations should be tested more routinely for promising materials formed using new or established synthesis routes. Ensuring transparency over testing conditions and open reporting of failure mechanisms is strongly encouraged. Testing a cell to failure rather than simply providing the longest stable cycle number (e.g., 100, 200, etc.) and targeted post-mortem analysis can significantly boost the knowledge gained from such testing.

The future prospects of Si NWs must factor in the context of cost and scalability of the new anodes on a $/KWh basis to ensure that the technology is commercially viable. While there may be scope for a slight premium compared to graphite (currently ca. 6 $/KWh), this should be the minimum target to ensure that the technology is competitive for widespread uptake.

As reported in this Perspective, there are several parameters which influence the performance of Si NW cells (e.g., electrolyte volume, N/P ratios, Si loading, electrode mass loadings, formation cycles, effect of voltage window, etc.). Therefore, the incorporation of machine learning techniques to predict appropriate recipes for the best performing configurations should be the next step to expedite the commercialization of Si NW-containing anodes.

Advanced characterization of Si NWs can shed light on SEI formation, structural evaluation, and dead Si formation. Si NWs represent an ideal model system for interrogating critical performance-related mechanisms related to Si and other alloying type anode materials. Further examinations using operando techniques (e.g., Nano-CT) should be carried out to allow NW-based anodes to be interrogated under practical conditions (i.e., within pouch cells). Many ex situ studies have previously used a large amount of electrolyte and excess Li as counter electrode (in half-cells) which differs substantially from the environment of a commercial pouch cell.

Si NWs have been scarcely used in beyond-LIB applications. Therefore, it is highly recommended that consideration should be given to this earth-abundant material and Si NW morphology in particular to harness its advantages in applications such as NIB and Li metal anodes. In the case of Na, if the capacity linked to the formation of NaSi can be unlocked, this would be a significant advance beyond hard-carbon anodes. For Li metal anode development, this offers the potential to drastically reduce the required Si loading to achieve commercially relevant areal capacities. This might represent the “best of both worlds”, where small mass loadings of Si NWs can be used to control Li deposition in practical Li anodes.

## References

[ref1] Li M.; Lu J.; Chen Z.; Amine K. 30 Years of Lithium-Ion Batteries. Adv. Mater. 2018, 30 (33), 1800561 10.1002/adma.201800561. 29904941

[ref2] Sharma R. A.; Seefurth R. N. Thermodynamic Properties of the Lithium-Silicon System. J. Electrochem. Soc. 1976, 123 (12), 1763–1768. 10.1149/1.2132692.

[ref3] Obrovac M. N.; Christensen L. Structural Changes in Silicon Anodes during Lithium Insertion/Extraction. Electrochem. Solid-State Lett. 2004, 7 (5), A93 10.1149/1.1652421.

[ref4] Mcdowell M. T.; Lee W.; Nix W. D.; Cui Y. 25th Anniversary Article: Understanding the Lithiation of Silicon and Other Alloying Anodes for Lithium-Ion Batteries. Adv. Mater. 2013, 25 (36), 4966–4985. 10.1002/adma.201301795. 24038172

[ref5] Jia H.; Li X.; Song J.; Zhang X.; Luo L.; He Y.; Li B.; Cai Y.; Hu S.; Xiao X.; Wang C.; Rosso K. M.; Yi R.; Patel R.; Zhang J. G. Hierarchical Porous Silicon Structures with Extraordinary Mechanical Strength as High-Performance Lithium-Ion Battery Anodes. Nature Communications 2020 11:1 2020, 11 (1), 1–9. 10.1038/s41467-020-15217-9. PMC7081208 32193387

[ref6] Liu N.; Lu Z.; Zhao J.; Mcdowell M. T.; Lee H. W.; Zhao W.; Cui Y. A Pomegranate-Inspired Nanoscale Design for Large-Volume-Change Lithium Battery Anodes. Nature Nanotechnology 2014 9:3 2014, 9 (3), 187–192. 10.1038/nnano.2014.6. 24531496

[ref7] Zhang X.; Shi H.; Lv P.; Liu J.; Zhang H. Engineering Nanostructured Silicon and Its Practical Applications in Lithium-Ion Batteries: A Critical Review. Energy Technology 2021, 9 (10), 2100400 10.1002/ente.202100400.

[ref8] Su X.; Wu Q.; Li J.; Xiao X.; Lott A.; Lu W.; Sheldon B. W.; Wu J. Silicon-Based Nanomaterials for Lithium-Ion Batteries: A Review. Adv. Energy Mater. 2014, 4 (1), 1300882 10.1002/aenm.201300882.

[ref9] Sun L.; Liu Y.; Shao R.; Wu J.; Jiang R.; Jin Z. Recent Progress and Future Perspective on Practical Silicon Anode-Based Lithium Ion Batteries. Energy Storage Mater. 2022, 46, 482–502. 10.1016/j.ensm.2022.01.042.

[ref10] Ge M.; Cao C.; Biesold G. M.; Sewell C. D.; Hao S.-M.; Huang J.; Zhang W.; Lai Y.; Lin Z. Recent Advances in Silicon-Based Electrodes: From Fundamental Research toward Practical Applications. Adv. Mater. 2021, 33 (16), 2004577 10.1002/adma.202004577. 33686697

[ref11] Wang F.; Chen G.; Zhang N.; Liu X.; Ma R. Engineering of Carbon and Other Protective Coating Layers for Stabilizing Silicon Anode Materials. Carbon Energy 2019, 1 (2), 219–245. 10.1002/cey2.24.

[ref12] Chan C. K.; Peng H.; Liu G.; McIlwrath K.; Zhang X. F.; Huggins R. A.; Cui Y. High-Performance Lithium Battery Anodes Using Silicon Nanowires. Nature Nanotechnology 2008 3:1 2008, 3 (1), 31–35. 10.1038/nnano.2007.411. 18654447

[ref13] Kennedy T.; Brandon M.; Laffir F.; Ryan K. M. Understanding the Influence of Electrolyte Additives on the Electrochemical Performance and Morphology Evolution of Silicon Nanowire Based Lithium-Ion Battery Anodes. J. Power Sources 2017, 359, 601–610. 10.1016/j.jpowsour.2017.05.093.

[ref14] Chan C. K.; Ruffo R.; Hong S. S.; Cui Y. Surface Chemistry and Morphology of the Solid Electrolyte Interphase on Silicon Nanowire Lithium-Ion Battery Anodes. J. Power Sources 2009, 189 (2), 1132–1140. 10.1016/j.jpowsour.2009.01.007.

[ref15] Zhang Q.; Zhang W.; Wan W.; Cui Y.; Wang E. Lithium Insertion in Silicon Nanowires: An Ab Initio Study. Nano Lett. 2010, 10 (9), 3243–3249. 10.1021/nl904132v. 20681548

[ref16] Gu M.; Parent L. R.; Mehdi B. L.; Unocic R. R.; McDowell M. T.; Sacci R. L.; Xu W.; Connell J. G.; Xu P.; Abellan P.; Chen X.; Zhang Y.; Perea D. E.; Evans J. E.; Lauhon L. J.; Zhang J. G.; Liu J.; Browning N. D.; Cui Y.; Arslan I.; Wang C. M. Demonstration of an Electrochemical Liquid Cell for Operando Transmission Electron Microscopy Observation of the Lithiation/Delithiation Behavior of Si Nanowire Battery Anodes. Nano Lett. 2013, 13 (12), 6106–6112. 10.1021/nl403402q. 24224495

[ref17] Cui L. F.; Yang Y.; Hsu C. M.; Cui Y. Carbon-Silicon Core-Shell Nanowires as High Capacity Electrode for Lithium Lon Batteries. Nano Lett. 2009, 9 (9), 3370–3374. 10.1021/nl901670t. 19655765

[ref18] Luo L.; Yang H.; Yan P.; Travis J. J.; Lee Y.; Liu N.; Molina Piper D.; Lee S. H.; Zhao P.; George S. M.; Zhang J. G.; Cui Y.; Zhang S.; Ban C.; Wang C. M. Surface-Coating Regulated Lithiation Kinetics and Degradation in Silicon Nanowires for Lithium Ion Battery. ACS Nano 2015, 9 (5), 5559–5566. 10.1021/acsnano.5b01681. 25893684

[ref19] Kennedy T.; Bezuidenhout M.; Palaniappan K.; Stokes K.; Brandon M.; Ryan K. M. Nanowire Heterostructures Comprising Germanium Stems and Silicon Branches as High-Capacity Li-Ion Anodes with Tunable Rate Capability. ACS Nano 2015, 9 (7), 7456–7465. 10.1021/acsnano.5b02528. 26125966

[ref20] Chan C. K.; Zhang X. F.; Cui Y. High Capacity Li Ion Battery Anodes Using Ge Nanowires. Nano Lett. 2008, 8 (1), 307–309. 10.1021/nl0727157. 18095738

[ref21] Kennedy T.; Mullane E.; Geaney H.; Osiak M.; O’Dwyer C.; Ryan K. M. High-Performance Germanium Nanowire-Based Lithium-Ion Battery Anodes Extending over 1000 Cycles through in Situ Formation of a Continuous Porous Network. Nano Lett. 2014, 14 (2), 716–723. 10.1021/nl403979s. 24417719

[ref22] Schmidt V.; Wittemann J. V.; Senz S.; Gósele U. Silicon Nanowires: A Review on Aspects of Their Growth and Their Electrical Properties. Adv. Mater. 2009, 21 (25–26), 2681–2702. 10.1002/adma.200803754. 36751058

[ref23] Yang Y.; Yuan W.; Kang W.; Ye Y.; Pan Q.; Zhang X.; Ke Y.; Wang C.; Qiu Z.; Tang Y. A Review on Silicon Nanowire-Based Anodes for next-Generation High-Performance Lithium-Ion Batteries from a Material-Based Perspective. Sustain Energy Fuels 2020, 4 (4), 1577–1594. 10.1039/C9SE01165J.

[ref24] Raman S.; Ravi Sankar A.; Sindhuja M. Advances in Silicon Nanowire Applications in Energy Generation, Storage, Sensing, and Electronics: A Review. Nanotechnology 2023, 34 (18), 182001 10.1088/1361-6528/acb320. 36640446

[ref25] Zamfir M. R.; Nguyen H. T.; Moyen E.; Lee Y. H.; Pribat D. Silicon Nanowires for Li-Based Battery Anodes: A Review. J. Mater. Chem. A Mater. 2013, 1 (34), 9566–9586. 10.1039/c3ta11714f.

[ref26] Schmidt V.; Wittemann J. V.; Gösele U. Growth, Thermodynamics, and Electrical Properties of Silicon Nanowires. Chem. Rev. 2010, 110 (1), 361–388. 10.1021/cr900141g. 20070117

[ref27] Kennedy T.; Brandon M.; Ryan K. M. Advances in the Application of Silicon and Germanium Nanowires for High-Performance Lithium-Ion Batteries. Adv. Mater. 2016, 28 (27), 5696–5704. 10.1002/adma.201503978. 26855084

[ref28] Aricò A. S.; Bruce P.; Scrosati B.; Tarascon J. M.; Van Schalkwijk W. Nanostructured Materials for Advanced Energy Conversion and Storage Devices. Nature Materials 2005 4:5 2005, 4 (5), 366–377. 10.1038/nmat1368. 15867920

[ref29] Chan C. K.; Patel R. N.; O’Connell M. J.; Korgel B. A.; Cui Y. Solution-Grown Silicon Nanowires for Lithium-Ion Battery Anodes. ACS Nano 2010, 4 (3), 1443–1450. 10.1021/nn901409q. 20201547

[ref30] Burchak O.; Keller C.; Lapertot G.; Salaün M.; Danet J.; Chen Y.; Bendiab N.; Pépin-Donat B.; Lombard C.; Faure-Vincent J.; Vignon A.; Aradilla D.; Reiss P.; Chenevier P. Scalable Chemical Synthesis of Doped Silicon Nanowires for Energy Applications. Nanoscale 2019, 11 (46), 22504–22514. 10.1039/C9NR03749G. 31746905

[ref31] Karuppiah S.; Keller C.; Kumar P.; Jouneau P. H.; Aldakov D.; Ducros J. B.; Lapertot G.; Chenevier P.; Haon C. A Scalable Silicon Nanowires-Grown-On-Graphite Composite for High-Energy Lithium Batteries. ACS Nano 2020, 14 (9), 12006–12015. 10.1021/acsnano.0c05198. 32902949

[ref32] Peled E.; Patolsky F.; Golodnitsky D.; Freedman K.; Davidi G.; Schneier D. Tissue-like Silicon Nanowires-Based Three-Dimensional Anodes for High-Capacity Lithium Ion Batteries. Nano Lett. 2015, 15 (6), 3907–3916. 10.1021/acs.nanolett.5b00744. 25970605

[ref33] Song H.; Wang S.; Song X.; Yang H.; Du G.; Yu L.; Xu J.; He P.; Zhou H.; Chen K. A Bottom-up Synthetic Hierarchical Buffer Structure of Copper Silicon Nanowire Hybrids as Ultra-Stable and High-Rate Lithium-Ion Battery Anodes. J. Mater. Chem. A Mater. 2018, 6 (17), 7877–7886. 10.1039/C8TA01694A.

[ref34] Collins G. A.; Kilian S.; Geaney H.; Ryan K. M. A Nanowire Nest Structure Comprising Copper Silicide and Silicon Nanowires for Lithium-Ion Battery Anodes with High Areal Loading. Small 2021, 17 (34), 2102333 10.1002/smll.202102333. 34263558

[ref35] Lu J.; Wu T.; Amine K. State-of-the-Art Characterization Techniques for Advanced Lithium-Ion Batteries. Nature Energy 2017 2:3 2017, 2 (3), 1–13. 10.1038/nenergy.2017.11.

[ref36] Stokes K.; Flynn G.; Geaney H.; Bree G.; Ryan K. M. Axial Si-Ge Heterostructure Nanowires as Lithium-Ion Battery Anodes. Nano Lett. 2018, 18 (9), 5569–5575. 10.1021/acs.nanolett.8b01988. 30091609

[ref37] He Y.; Jiang L.; Chen T.; Xu Y.; Jia H.; Yi R.; Xue D.; Song M.; Genc A.; Bouchet-Marquis C.; Pullan L.; Tessner T.; Yoo J.; Li X.; Zhang J. G.; Zhang S.; Wang C. Progressive Growth of the Solid–Electrolyte Interphase towards the Si Anode Interior Causes Capacity Fading. Nature Nanotechnology 2021 16:10 2021, 16 (10), 1113–1120. 10.1038/s41565-021-00947-8. 34326526

[ref38] Bareño J.; Shkrob I. A.; Gilbert J. A.; Klett M.; Abraham D. P. Capacity Fade and Its Mitigation in Li-Ion Cells with Silicon-Graphite Electrodes. J. Phys. Chem. C 2017, 121 (38), 20640–20649. 10.1021/acs.jpcc.7b06118.

[ref39] Fang C.; Li J.; Zhang M.; Zhang Y.; Yang F.; Lee J. Z.; Lee M. H.; Alvarado J.; Schroeder M. A.; Yang Y.; Lu B.; Williams N.; Ceja M.; Yang L.; Cai M.; Gu J.; Xu K.; Wang X.; Meng Y. S. Quantifying Inactive Lithium in Lithium Metal Batteries. Nature 2019 572:7770 2019, 572 (7770), 511–515. 10.1038/s41586-019-1481-z. 31435056

[ref40] Bao W.; Fang C.; Cheng D.; Zhang Y.; Lu B.; Tan D. H. S.; Shimizu R.; Sreenarayanan B.; Bai S.; Li W.; Zhang M.; Meng Y. S. Quantifying Lithium Loss in Amorphous Silicon Thin-Film Anodes via Titration-Gas Chromatography. Cell Rep. Phys. Sci. 2021, 2 (10), 100597 10.1016/j.xcrp.2021.100597.

[ref41] Zhao C.; Wada T.; De Andrade V.; Gürsoy D.; Kato H.; Chen-Wiegart Y. Imaging of 3D Morphological Evolution of Nanoporous Silicon Anode in Lithium Ion Battery by X-Ray Nano-Tomography. Nano Energy 2018, 52, 381–390. 10.1016/j.nanoen.2018.08.009.

[ref42] Jeong H.; Jang J.; Jo C. A Review on Current Collector Coating Methods for Next-Generation Batteries. Chemical Engineering Journal 2022, 446, 136860 10.1016/j.cej.2022.136860.

[ref43] Hamon Y.; Brousse T.; Jousse F.; Topart P.; Buvat P.; Schleich D. M. Aluminum Negative Electrode in Lithium Ion Batteries. J. Power Sources 2001, 97–98, 185–187. 10.1016/S0378-7753(01)00616-4.

[ref44] Fear C.; Juarez-Robles D.; Jeevarajan J. A.; Mukherjee P. P. Elucidating Copper Dissolution Phenomenon in Li-Ion Cells under Overdischarge Extremes. J. Electrochem. Soc. 2018, 165 (9), A1639–A1647. 10.1149/2.0671809jes.

[ref45] Zhu P.; Gastol D.; Marshall J.; Sommerville R.; Goodship V.; Kendrick E. A Review of Current Collectors for Lithium-Ion Batteries. J. Power Sources 2021, 485, 229321 10.1016/j.jpowsour.2020.229321.

[ref46] Geaney H.; Dickinson C.; O’Dwyer C.; Mullane E.; Singh A.; Ryan K. M. Growth of Crystalline Copper Silicide Nanowires in High Yield within a High Boiling Point Solvent System. Chem. Mater. 2012, 24 (22), 4319–4325. 10.1021/cm302066n.

[ref47] Imtiaz S.; Amiinu I. S.; Storan D.; Kapuria N.; Geaney H.; Kennedy T.; Ryan K. M. Dense Silicon Nanowire Networks Grown on a Stainless-Steel Fiber Cloth: A Flexible and Robust Anode for Lithium-Ion Batteries. Adv. Mater. 2021, 33 (52), 2105917 10.1002/adma.202105917. PMC11469259 34613631

[ref48] Kilian S.; McCarthy K.; Stokes K.; Adegoke T. E.; Conroy M.; Amiinu I. S.; Geaney H.; Kennedy T.; Ryan K. M. Direct Growth of Si, Ge, and Si–Ge Heterostructure Nanowires Using Electroplated Zn: An Inexpensive Seeding Technique for Li-Ion Alloying Anodes. Small 2021, 17 (10), 2005443 10.1002/smll.202005443. 33475259

[ref49] Geaney H.; Mullane E.; Ramasse Q. M.; Ryan K. M. Atomically Abrupt Silicon-Germanium Axial Heterostructure Nanowires Synthesized in a Solvent Vapor Growth System. Nano Lett. 2013, 13 (4), 1675–1680. 10.1021/nl400146u. 23517564

[ref50] Geaney H.; Kennedy T.; Dickinson C.; Mullane E.; Singh A.; Laffir F.; Ryan K. M. High Density Growth of Indium Seeded Silicon Nanowires in the Vapor Phase of a High Boiling Point Solvent. Chem. Mater. 2012, 24 (11), 2204–2210. 10.1021/cm301023j.

[ref51] Barrett C. A.; Geaney H.; Gunning R. D.; Laffir F. R.; Ryan K. M. Perpendicular Growth of Catalyst-Free Germanium Nanowire Arrays. Chem. Commun. 2011, 47 (13), 3843–3845. 10.1039/c0cc05202g. 21321699

[ref52] Rashad M.; Geaney H. Vapor-Solid-Solid Growth of Silicon Nanowires Using Magnesium Seeds and Their Electrochemical Performance in Li-Ion Battery Anodes. Chemical Engineering Journal 2023, 452, 139397 10.1016/j.cej.2022.139397.

[ref53] Zhou H.; Nanda J.; Martha S. K.; Unocic R. R.; Meyer H. M.; Sahoo Y.; Miskiewicz P.; Albrecht T. F. Role of Surface Functionality in the Electrochemical Performance of Silicon Nanowire Anodes for Rechargeable Lithium Batteries. ACS Appl. Mater. Interfaces 2014, 6 (10), 7607–7614. 10.1021/am500855a. 24731257

[ref54] Aminu I. S.; Geaney H.; Imtiaz S.; Adegoke T. E.; Kapuria N.; Collins G. A.; Ryan K. M. A Copper Silicide Nanofoam Current Collector for Directly Grown Si Nanowire Networks and Their Application as Lithium-Ion Anodes. Adv. Funct Mater. 2020, 30 (38), 2003278 10.1002/adfm.202003278.

[ref55] Baasner A.; Reuter F.; Seidel M.; Krause A.; Pflug E.; Härtel P.; Dörfler S.; Abendroth T.; Althues H.; Kaskel S. The Role of Balancing Nanostructured Silicon Anodes and NMC Cathodes in Lithium-Ion Full-Cells with High Volumetric Energy Density. J. Electrochem. Soc. 2020, 167 (2), 020516 10.1149/1945-7111/ab68d7.

[ref56] Wang H.; Song H.; Lin Z.; Jiang X.; Zhang X.; Yu L.; Xu J.; Pan L.; Wang J.; Zheng M.; Shi Y.; Chen K. Highly Cross-Linked Cu/a-Si Core–Shell Nanowires for Ultra-Long Cycle Life and High Rate Lithium Batteries. Nanoscale 2016, 8 (5), 2613–2619. 10.1039/C5NR06985H. 26572901

[ref57] Zhang Z.; Wang Z. L.; Lu X. Multishelled Si@Cu Microparticles Supported on 3D Cu Current Collectors for Stable and Binder-Free Anodes of Lithium-Ion Batteries. ACS Nano 2018, 12 (4), 3587–3599. 10.1021/acsnano.8b00703. 29630825

[ref58] Stokes K.; Geaney H.; Sheehan M.; Borsa D.; Ryan K. M. Copper Silicide Nanowires as Hosts for Amorphous Si Deposition as a Route to Produce High Capacity Lithium-Ion Battery Anodes. Nano Lett. 2019, 19 (12), 8829–8835. 10.1021/acs.nanolett.9b03664. 31671264

[ref59] Ryou M. H.; Kim S. H.; Kim S. W.; Lee S. Y. A Microgrid-Patterned Silicon Electrode as an Electroactive Lithium Host. Energy Environ. Sci. 2022, 15 (6), 2581–2590. 10.1039/D2EE00981A.

[ref60] Li X.; Yan P.; Xiao X.; Woo J. H.; Wang C.; Liu J.; Zhang J. G. Design of Porous Si/C–Graphite Electrodes with Long Cycle Stability and Controlled Swelling. Energy Environ. Sci. 2017, 10 (6), 1427–1434. 10.1039/C7EE00838D.

[ref61] Li P.; Hwang J. Y.; Sun Y. K. Nano/Microstructured Silicon-Graphite Composite Anode for High-Energy-Density Li-Ion Battery. ACS Nano 2019, 13 (2), 2624–2633. 10.1021/acsnano.9b00169. 30759341

[ref62] Zhu S.; Zhou J.; Guan Y.; Cai W.; Zhao Y.; Zhu Y.; Zhu L.; Zhu Y.; Qian Y. Hierarchical Graphene-Scaffolded Silicon/Graphite Composites as High Performance Anodes for Lithium-Ion Batteries. Small 2018, 14 (47), 1802457 10.1002/smll.201802457. 30328267

[ref63] Li X.; Colclasure A. M.; Finegan D. P.; Ren D.; Shi Y.; Feng X.; Cao L.; Yang Y.; Smith K. Degradation Mechanisms of High Capacity 18650 Cells Containing Si-Graphite Anode and Nickel-Rich NMC Cathode. Electrochim. Acta 2019, 297, 1109–1120. 10.1016/j.electacta.2018.11.194.

[ref64] Wang W.; Wang Y.; Yuan L.; You C.; Wu J.; Liu L.; Ye J.; Wu Y.; Fu L. Recent Advances in Modification Strategies of Silicon-Based Lithium-Ion Batteries. Nano Research 2022 16:3 2023, 16 (3), 3781–3803. 10.1007/s12274-022-5147-z.

[ref65] An S. J.; Li J.; Daniel C.; Meyer H. M.; Trask S. E.; Polzin B. J.; Wood D. L. Electrolyte Volume Effects on Electrochemical Performance and Solid Electrolyte Interphase in Si-Graphite/NMC Lithium-Ion Pouch Cells. ACS Appl. Mater. Interfaces 2017, 9 (22), 18799–18808. 10.1021/acsami.7b03617. 28505406

[ref66] Harper G.; Sommerville R.; Kendrick E.; Driscoll L.; Slater P.; Stolkin R.; Walton A.; Christensen P.; Heidrich O.; Lambert S.; Abbott A.; Ryder K.; Gaines L.; Anderson P. Recycling Lithium-Ion Batteries from Electric Vehicles. Nature 2019 575:7781 2019, 575 (7781), 75–86. 10.1038/s41586-019-1682-5. 31695206

[ref67] Zheng P.; Young D.; Yang T.; Xiao Y.; Li Z. Powering Battery Sustainability: A Review of the Recent Progress and Evolving Challenges in Recycling Lithium-Ion Batteries. Frontiers in Sustainable Resource Management 2023, 2, 1127001 10.3389/fsrma.2023.1127001.

[ref68] Lu J.; Liu S.; Liu J.; Qian G.; Wang D.; Gong X.; Deng Y.; Chen Y.; Wang Z. Millisecond Conversion of Photovoltaic Silicon Waste to Binder-Free High Silicon Content Nanowires Electrodes. Adv. Energy Mater. 2021, 11 (40), 2102103 10.1002/aenm.202102103.

[ref69] Jeong Y. K.; Huang W.; Vilá R. A.; Huang W.; Wang J.; Cheol Kim S.; Seok Kim Y.; Zhao J.; Cui Y. Microclusters of Kinked Silicon Nanowires Synthesized by a Recyclable Iodide Process for High-Performance Lithium-Ion Battery Anodes. Adv. Energy Mater. 2020, 10 (41), 2002108 10.1002/aenm.202002108.

[ref70] Zhu Y.; Pluvinage V. Manufacturing Apparatus and Method for Making Silicon Nanowires on Carbon Based Powders for Use in Batteries. US Patent 10862114B2, July 14, 2017.

[ref71] Zhu Y.; Du C. Du; Shin J. Silicon Nanostructure Active Materials for Lithium Ion Batteries and Processes, Compositions, Components, and Devices Related Thereto. US Patent 9812699B2, July 16, 2019.

[ref72] Cao W.; Robbins V.; Zhu Y. Nanostructured Battery Active Materials and Methods of Producing Same, US Patent 10243207B2, July 24, 2012.

[ref73] OneD Battery Sciences. The key to silicon anode solutions: Cost. https://onedsinanode.com/media-room/news-and-press/the-key-to-silicon-anode-solutions-cost/ (accessed 2024-01-20).

[ref74] Sila Nanotechnologies Inc. A Market Overview of Anode Materials: Designing A Better Battery for Next Generation EVs; 2023. https://www.silanano.com/insights/a-market-overview-of-anode-materials (accessed 2024-01-20).

[ref75] Abdul Ahad S.; Bhattacharya S.; Kilian S.; Ottaviani M.; Ryan K. M.; Kennedy T.; Thompson D.; Geaney H. Lithiophilic Nanowire Guided Li Deposition in Li Metal Batteries. Small 2023, 19 (2), 2205142 10.1002/smll.202205142. 36398602

[ref76] Zhang P.; Peng C.; Liu X.; Dong F.; Xu H.; Yang J.; Zheng S. 3D Lithiophilic “Hairy” Si Nanowire Arrays @ Carbon Scaffold Favor a Flexible and Stable Lithium Composite Anode. ACS Appl. Mater. Interfaces 2019, 11 (47), 44325–44332. 10.1021/acsami.9b15250. 31674757

[ref77] Grandjean M.; Pichardo M.; Biecher Y.; Haon C.; Chenevier P. Matching Silicon-Based Anodes with Sulfide-Based Solid-State Electrolytes for Li-Ion Batteries. J. Power Sources 2023, 580, 233386 10.1016/j.jpowsour.2023.233386.

[ref78] Cangaz S.; Hippauf F.; Reuter F. S.; Doerfler S.; Abendroth T.; Althues H.; Kaskel S. Enabling High-Energy Solid-State Batteries with Stable Anode Interphase by the Use of Columnar Silicon Anodes. Adv. Energy Mater. 2020, 10 (34), 2001320 10.1002/aenm.202001320.

[ref79] Abdul Ahad S.; Kilian S.; Zubair M.; Lebedev V. A.; McNamara K.; Ryan K. M.; Kennedy T.; Geaney H. Amorphization Driven Na-Alloying in SixGe1–x Alloy Nanowires for Na-Ion Batteries. J. Mater. Chem. A Mater. 2021, 9 (36), 20626–20634. 10.1039/D1TA03741B.

[ref80] Jangid M. K.; Lakhnot A. S.; Vemulapally A.; Sonia F. J.; Sinha S.; Dusane R. O.; Mukhopadhyay A. Crystalline Core/Amorphous Shell Structured Silicon Nanowires Offer Size and Structure Dependent Reversible Na-Storage. J. Mater. Chem. A Mater. 2018, 6 (8), 3422–3434. 10.1039/C7TA10249F.

[ref81] Wang Q.; Liu B.; Shen Y.; Wu J.; Zhao Z.; Zhong C.; Hu W. Confronting the Challenges in Lithium Anodes for Lithium Metal Batteries. Advanced Science 2021, 8 (17), 2101111 10.1002/advs.202101111. 34196478 PMC8425877

[ref82] Yan K.; Lu Z.; Lee H. W.; Xiong F.; Hsu P. C.; Li Y.; Zhao J.; Chu S.; Cui Y. Selective Deposition and Stable Encapsulation of Lithium through Heterogeneous Seeded Growth. Nature Energy 2016 1:3 2016, 1 (3), 1–8. 10.1038/nenergy.2016.10.

[ref83] Abdul Ahad S.; Adegoke T. E.; Ryan K. M.; Geaney H. Cu Current Collector with Binder-Free Lithiophilic Nanowire Coating for High Energy Density Lithium Metal Batteries. Small 2023, 19 (20), 2207902 10.1002/smll.202207902. 36802164

[ref84] Jin S.; Ye Y.; Niu Y.; Xu Y.; Jin H.; Wang J.; Sun Z.; Cao A.; Wu X.; Luo Y.; Ji H.; Wan L. J. Solid-Solution-Based Metal Alloy Phase for Highly Reversible Lithium Metal Anode. J. Am. Chem. Soc. 2020, 142 (19), 8818–8826. 10.1021/jacs.0c01811. 32310653

[ref85] Wang H.; Cao X.; Gu H.; Liu Y.; Li Y.; Zhang Z.; Huang W.; Wang H.; Wang J.; Xu W.; Zhang J. G.; Cui Y. Improving Lithium Metal Composite Anodes with Seeding and Pillaring Effects of Silicon Nanoparticles. ACS Nano 2020, 14 (4), 4601–4608. 10.1021/acsnano.0c00184. 32271533

[ref86] Zhang Y.; Wang C.; Pastel G.; Kuang Y.; Xie H.; Li Y.; Liu B.; Luo W.; Chen C.; Hu L. 3D Wettable Framework for Dendrite-Free Alkali Metal Anodes. Adv. Energy Mater. 2018, 8 (18), 1800635 10.1002/aenm.201800635.

[ref87] Lu L. L.; Ge J.; Yang J. N.; Chen S. M.; Yao H.-B.; Zhou F.; Yu S. H. Free-Standing Copper Nanowire Network Current Collector for Improving Lithium Anode Performance. Nano Lett. 2016, 16 (7), 4431–4437. 10.1021/acs.nanolett.6b01581. 27253417

[ref88] Yang C. P.; Yin Y. X.; Zhang S. F.; Li N. W.; Guo Y. G. Accommodating Lithium into 3D Current Collectors with a Submicron Skeleton towards Long-Life Lithium Metal Anodes. Nature Communications 2015 6:1 2015, 6 (1), 1–9. 10.1038/ncomms9058. PMC4560781 26299379

[ref89] Na I.; Kim H.; Kunze S.; Nam C.; Jo S.; Choi H.; Oh S.; Choi E.; Song Y. B.; Jung Y. S.; Lee Y. S.; Lim J. Monolithic 100% Silicon Wafer Anode for All-Solid-State Batteries Achieving High Areal Capacity at Room Temperature. ACS Energy Lett. 2023, 8 (4), 1936–1943. 10.1021/acsenergylett.3c00496.

[ref90] Huang Y.; Shao B.; Wang Y.; Han F. Solid-State Silicon Anode with Extremely High Initial Coulombic Efficiency. Energy Environ. Sci. 2023, 16 (4), 1569–1580. 10.1039/D2EE04057C.

[ref91] Tan D. H. S.; Chen Y. T.; Yang H.; Bao W.; Sreenarayanan B.; Doux J. M.; Li W.; Lu B.; Ham S. Y.; Sayahpour B.; Scharf J.; Wu E. A.; Deysher G.; Han H. E.; Hah H. J.; Jeong H.; Lee J. B.; Chen Z.; Meng Y. S. Carbon-Free High-Loading Silicon Anodes Enabled by Sulfide Solid Electrolytes. Science (1979) 2021, 373 (6562), 1494–1499. 10.1126/science.abg7217. 34554780

[ref92] Ohta N.; Kimura S.; Sakabe J.; Mitsuishi K.; Ohnishi T.; Takada K. Anode Properties of Si Nanoparticles in All-Solid-State Li Batteries. ACS Appl. Energy Mater. 2019, 2 (10), 7005–7008. 10.1021/acsaem.9b01517.

[ref93] Sakabe J.; Ohta N.; Ohnishi T.; Mitsuishi K.; Takada K. Porous Amorphous Silicon Film Anodes for High-Capacity and Stable All-Solid-State Lithium Batteries. Communications Chemistry 2018 1:1 2018, 1 (1), 1–9. 10.1038/s42004-018-0026-y.

[ref94] Miyazaki R.; Ohta N.; Ohnishi T.; Sakaguchi I.; Takada K. An Amorphous Si Film Anode for All-Solid-State Lithium Batteries. J. Power Sources 2014, 272, 541–545. 10.1016/j.jpowsour.2014.08.109.

[ref95] Usiskin R.; Lu Y.; Popovic J.; Law M.; Balaya P.; Hu Y. S.; Maier J. Fundamentals, Status and Promise of Sodium-Based Batteries. Nature Reviews Materials 2021 6:11 2021, 6 (11), 1020–1035. 10.1038/s41578-021-00324-w.

[ref96] Shao W.; Shi H.; Jian X.; Wu Z. S.; Hu F. Hard-Carbon Anodes for Sodium-Ion Batteries: Recent Status and Challenging Perspectives. Advanced Energy and Sustainability Research 2022, 3 (7), 2200009 10.1002/aesr.202200009.

[ref97] Legrain F.; Malyi O. I.; Manzhos S. Comparative Computational Study of the Energetics of Li, Na, and Mg Storage in Amorphous and Crystalline Silicon. Comput. Mater. Sci. 2014, 94 (C), 214–217. 10.1016/j.commatsci.2014.04.010.

[ref98] Jung S. C.; Jung D. S.; Choi J. W.; Han Y. K. Atom-Level Understanding of the Sodiation Process in Silicon Anode Material. J. Phys. Chem. Lett. 2014, 5 (7), 1283–1288. 10.1021/jz5002743. 26274485

[ref99] Chou C. Y.; Lee M.; Hwang G. S. A Comparative First-Principles Study on Sodiation of Silicon, Germanium, and Tin for Sodium-Ion Batteries. J. Phys. Chem. C 2015, 119 (27), 14843–14850. 10.1021/acs.jpcc.5b01099.

[ref100] Sayed S. Y.; Kalisvaart W. P.; Luber E. J.; Olsen B. C.; Buriak J. M. Stabilizing Tin Anodes in Sodium-Ion Batteries by Alloying with Silicon. ACS Appl. Energy Mater. 2020, 3 (10), 9950–9962. 10.1021/acsaem.0c01641.

